# Normocalcemic primary hyperparathyroidism is an early stage of primary hyperparathyroidism according to fibroblast growth factor 23 level

**DOI:** 10.3389/fendo.2023.1152464

**Published:** 2023-03-30

**Authors:** Elena Chertok Shacham, Nimra Maman, Tatyana Lazareva, Refaat Masalha, Lila Mahagna, Gala Sela, Avraham Ishay

**Affiliations:** ^1^ Endocrinology Unit, Haemek Medical Center, Afula, Israel; ^2^ Statistical Department, Haemek Medical Center, Afula, Israel; ^3^ Internal Medicine Department A, Haemek Medical Center, Afula, Israel; ^4^ Laboratory Medicine Department, Haemek Medical Center, Afula, Israel; ^5^ Faculty of Medicine, Technion – Israel Institute of Technology, Haifa, Israel

**Keywords:** fibroblast growth factor 23, normocalcemic primary hyperparathyroidism, primary hyperparathyroidism, bone metabolism, calcium and phosphorus homeostasis

## Abstract

**Introduction:**

Normocalcemic primary hyperparathyroidism is a variant of primary hyperparathyroidism with consistently normal albumin-adjusted or free-ionized calcium levels. It may be an early stage of classic primary hyperparathyroidism or could represent primary kidney or bone disorder characterized by permanent elevation of PTH level.

**Aim of the study:**

The study aims to compare the FGF-23 levels in patients with PHPT, NPHPT, and normal calcium and PTH levels.

**Methods:**

Our study included patients who were referred to the endocrinology clinic with a presumptive diagnosis of primary hyperparathyroidism, an isolated increased level of PTH, or reduced bone densitometry. For each patient, we performed blood analysis of FGF-23, calcium, phosphate, vitamin D [25(OH)D3], estimated glomerular filtration rate (eGFR), bone turnover markers, and urine analysis for calcium/creatinine ratio.

**Results:**

Our study included 105 patients. Thirty patients with hypercalcemic hyperparathyroidism (HPHPT group), thirty patients with elevated PTH and normal calcium levels (NPHPT group), and 45 patients with normal calcium and PTH levels in the control group. FGF 23 level was 59.5± 23 pg/ml in the NPHPT group, 77 ± 33 pg/ml in the HPHPT group, and 49.7 ± 21.7 pg/ml in the control group (p=0.012). The phosphate level was lowest in the HPHPT group: 2.9 ± 0.6 vs 3.5 ± 0.44 in the NPHPT and 3.8 ± 0.5 in the control groups (p=0.001). No differences were found in eGFR, 25(OH)D3, C-terminal telopeptide type I collagen (CTX) and procollagen type 1 N-terminal propeptide (P1NP) levels, and bone densitometry scores between the three study groups.

**Conclusion:**

Our findings suggest that NPHPT is an early stage of PHPT. Further studies are needed to determine the role of FGF-23 and its usefulness in NPHPT.

## Introduction

Calcium and phosphate homeostasis is regulated by three key hormones: parathyroid hormone (PTH), vitamin D, and fibroblast growth factor (FGF)-23 (1). FGF-23 is a protein secreted by mature osteoblasts and osteocytes (2). In the kidney, FGF-23 binds to the FGFR1 receptor, and in presence of its co-receptor klotho, downregulates sodium phosphate type II and type III co-transporters. FGF-23 decreases 1,25(OH)_2_D synthesis in proximal tubules by direct inhibition of 1α-hydroxylase enzyme and reduces intestinal phosphate absorption (1, 2). Furthermore, in distal renal tubules, similarly to PTH, FGF23 increases the epithelial calcium channel transient receptor potential vanilloid-5 (TRPV5) mediated calcium reabsorption (3). The counter-regulatory effect of FGF-23 on the bone-kidney axis in chronic renal failure protects against hyperphosphatemia, vitamin D toxicity, and related soft-tissue calcifications (4, 5). Increased FGF-23 level was found in hereditary hypophosphatemic rickets and tumor-induced osteomalacia (6–8). Elevated FGF-23 level was found to be an independent risk for fragility fractures in elderly patients as well as in patients with chronic renal failure (9–11). FGF-23 levels were found elevated in patients with PHPT and correlated positively with serum calcium and PTH and negatively with GFR and 1,25(OH)_2_D3 (12). Normocalcemic PHPT is defined by persistently elevated PTH levels in the presence of consistently normal albumin-corrected and ionized calcium levels after excluding other causes of PTH elevation (13). The pathophysiology of NPHPT is still not elucidated. The most accepted concept is that it is an early form of the classic PHPT (13, 14) Nevertheless, it is not clear why some patients remain normocalcemic and others progress toward typical PHPT with time. It was suggested that the maintenance of normal ionized serum calcium in some patients with PHPT could be due to partial resistance to PTH in bone and kidney as well as calcium-sensing receptor (CaSR) polymorphism (14, 15). The purpose of our study is to investigate the FGF-23 levels in patients with NPHPT and HPHPT and in patients with normal calcium and PTH levels.

## Methods

### Design

It was a prospective observational study performed at the Endocrine Unit of Haemek Medical center, Afula, Israel (EMC 0139-18).

### Patients

We enrolled patients who were referred to the endocrinology clinic with a diagnosis of primary hyperparathyroidism, osteoporosis, or elevated PTH level with normal serum calcium between January 2020 and June 2022.

We finally enrolled 105 patients after excluding patients with malignancy, renal disease (estimated glomerular filtration rate < 60mL/min/1.73m^2^), and low 25(OH)D levels (< 60 nmol/L). The patients were divided into three groups according to repeated (at least 3 measurements over 6 months) serum-corrected calcium and PTH levels: the HPHPT group (elevated PTH and calcium levels), the NPHPT group (elevated PTH levels with normal calcium levels), and the control group (normal PTH and calcium levels). The study protocol was approved by the Institutional Review Board of our hospital. Informed consent was taken from each patient on the day of the laboratory investigations (index day).

### Biochemical analysis

Blood was drawn on the index day at 9-11 a.m. for FGF-23 level, calcium, phosphate, PTH, 25-hydroxyvitamin D (25(OH)D), and creatinine levels. The urine calcium/creatinine ratio was also assessed. FGF 23 level was measured by chemiluminescent immunoassays (Liaison systems). The reference range for this assay as assessed in 910 healthy controls ranged from 23.2 – 95.4 pg/mL. The laboratory CV for this assay is 5.8% to 7.1% at 18.2-2687.6 pg/ml. Intact parathyroid hormone was measured by the Advia Centaur XP immunoassay system. Vitamin D (25OH D) was measured by the Immunoassay system Advia Centaur XP (Siemens). CV ≤ 8% with the samples >20 ng/ml. Serum calcium was measured using an automated clinical chemistry analyzer (Beckman Coulter). Albumin measurement was performed using a Beckman Coulter analyzer. The albumin-adjusted calcium was used for this study.

The equation used to calculate eGFR was the Chronic Kidney Disease Epidemiology Collaboration (CKD-EPI).

### BMD, bone turnover markers

Data about BMD were retrieved from electronic charts of patients if it was performed up to 12 months before the index day otherwise, we refer the patients to perform DXA of the lumbar spine and the proximal femur in posteroanterior projection in a single bone densitometry center. The bone turnover markers, CTX, and P1NP were retrieved from electronic charts of patients if performed within 3 months of the index day.

### Osteoporosis treatment

We recorded all medications for osteoporosis treatment from the electronic charts of patients. Patients who refilled oral or intravenous bisphosphonates 12 months or denosumab 6 months before the index day were considered as treated for osteoporosis.

### Statistical analysis

The data were presented using mean, standard deviation, and median. A comparison between the study groups (patients with a normocalcemic and hypercalcemic group with high PTH levels and a control group with normal calcium and PTH) was performed by ANOVA test and the source of the difference between them was tested using the Tukey HSD method.

Prevalence and relative prevalence (%) was presented for categorical variables and the chi-square test was used for categorical variables and the Fisher Exact Test was used for non-parametric variables. A linear relationship between continuous variables - the (FGF23 value, PTH, and GFR rate) was examined using Pearson and Spearman correlation coefficients according to the existence of assumptions.

The difference in PTH level between the treatment groups (treatment 0/1) was evaluated by T-test for independent groups. Data analysis was performed using the statistical software IBM SPSS Statistics V 24. Statistical significance was obtained when p-VALUE<0.05. Parametric tests give a good approximation with a sufficient sample size.

## Results

Of the one hundred and five patients included in our study, 30 were assigned to the HPHPT group, 30 to the NPHPT group, and 45 to the group with normal calcium and PTH level (NPTH group). All but seven patients were women. Patients with a normal parathyroid hormone level had significantly lower BMI and systemic blood pressure compared to the two other study groups (**Table 1**). Patients with overt hyperparathyroidism (HPHPT group) had significantly elevated levels of calcium and parathyroid hormone (p=0.00). FGF 23 level was highest in the hypercalcemic group and lowest in the NPTH group (p=0.000) (**Table 2**). No significant differences were found between 25 OH vitamin D levels and eGFR in the three study groups (p=0.88 and 0.17, respectively). The urine calcium/creatine ratio was significantly higher in the HPHPT group compared to the two other groups (p=0.01) (**Table 2**). No significant differences were found in the bone turnover markers. No significant differences were found in the bone densitometry level in the spine or hip between the three study groups (**Table 2**).

**Table 1:** Clinical and anthropomorphic data.

Data	Total N=105	Normocalcemic PHPT N=30	PHPT N=30	Normocalcemic with normal PTH N=45	p-value
Age	69±7.9	69.7±7.2	69.3±9.1	68.4±7.7	0.77
Female sex (N)	98	28	27	43	0.08
BMI (kg/m²)	26.9±4.3	28.8±5.3	27.9±.3.9	25±3.2	0.00
Systolic blood pressure (mm Hg)	130±18	134±19	134±19	125±15	0.03
Diastolic blood pressure (mm Hg)	72±10	72±9	76±8	70±11	0.06
Previous fractures (10 years, N)	28	8	2	18	0.06

**Table 2:** Biochemical data and bone densitometry parameters.

Data	Total N=105	NPHPT N=30	PHPT N=30	NPTH N=45	p-value
Calcium (mg/dl)	9.9±0.8	9.5±0.4	10.9±0.5	9.4±0.3	0.00
Phosphor mg/d	3.5±0.6	3.4±0.4	2.9±0.6	3.8±0.5	0.00
PTH pg/ml	107±89	129±55	164±131[122]	55±16	0.000
FGF23 pg/ml	59.6±28.4	59.6±23.8	77±33	47.9±22	0.000
GFR (ml/min)	84±17	79±14	84±19	87±17	0.17
25 OH Vitamin D (nmol/l)	87±29	85±19	89±37	86±28	0.88
Urine calcium/creat ratio	0.130.18±	0.14±0.1	0.25±0.19	0.15±0.08	0.01
CTX (N=68) Mean, median, SD	0.33±0.25 (0.28)	0.3±0.22 (0.26)	0.46±0.33 (0.6)	0.31±0.24 (0.27)	0.37
P1NP (N=66) Mean, median, SD	42.4±23 (0.37)	42.5±28 (0.36)	51±24.6 (0.5)	41±20 (0.38)	0.62
BMD hip (g/cm²)	0.772±0.1	0.777±0.1	0.795±0.14	0.75±0.088	0.313
T score hip	2.2±0.7	-2.1±0.66	-2.2±0.88	-2.2±0.49	0.6
BMD spine (g/cm²)	-0.95±0.15	-0.97±0.13	-0.99±0.16	-0.92± 0.14	0.13
T score spine	-2.0±0.9	-1.9±0.9	-1.7±1	-2.3±0.9	0.04

*For non-normally distributed variables (P1NP and CTX) we add a median value.

### Correlations

**Figure 1:**
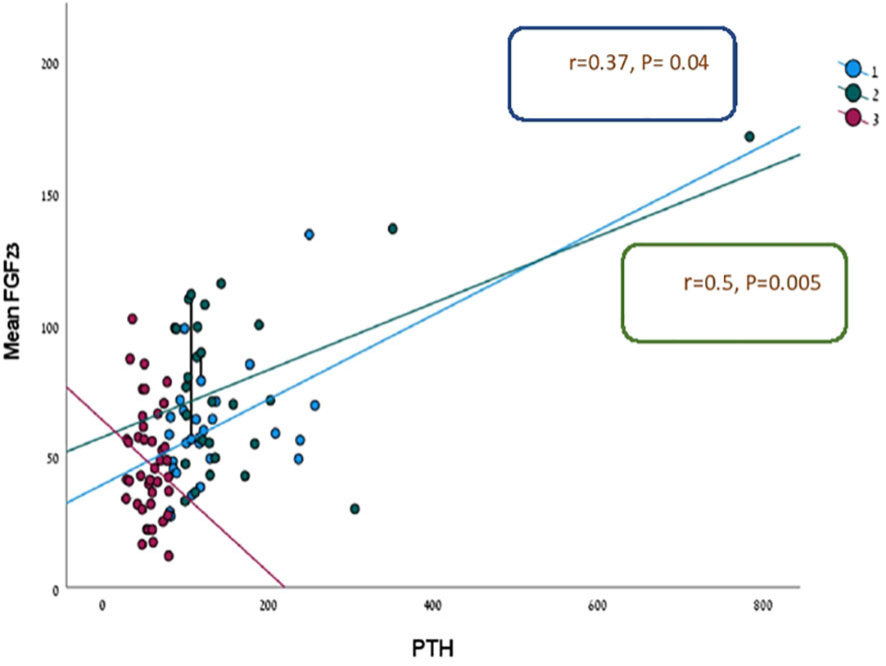
Linear regression analysis for prediction FGF-23 level according to PTH in three study groups.

**Figure 2:**
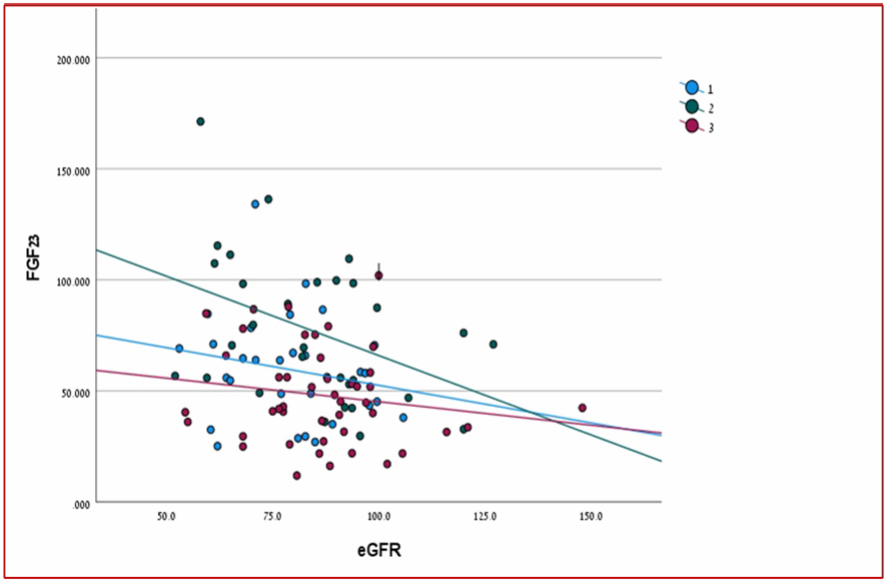
Relation of FGF-23 to eGFR in the three study groups.

There was a significant positive relationship between PTH and FGF23 levels in two out of three groups of patients (HPHPT and NPHPT groups) when all three groups of patients were analyzed together (p= 0.000) (**Figure 1**). A significant negative correlation was found between FGF-23 level and eGFR when all study groups were analyzed together (p=0.023) (**Figure 2**). Significant negative correlations were found between the FGF-23 level and the level of phosphate (p= 0.02). No significant correlations were found between calcium, vitamin 25OH D, and FGF-23 levels. The bone markers levels and bone densitometry measurements were similar in the 3 groups. However, a significant negative correlation was found between the T score at the spine and FGF23 levels. No significant correlations were found between the CTX and P1NP levels and FGF-23 concentration. PTH level was not found to be correlated with osteoporosis treatment.

### Osteoporosis treatment

Forty-seven out of 105 patients were treated with antiresorptive therapy. Among treated patients, 36 received oral or intravenous bisphosphonate, and 11 denosumab therapy (of whom four patients were in the NPHPT group). Four patients were treated with anabolic therapy (2 with teriparatide and 2 with romososumab). Previous fractures were reported in 28 out of 105 patients (nine vertebral, twenty-three radius and ribs, and five hip fractures), with 18 fractures occurring in the NPHPT group. Eighteen patients had multiple fractures (fourteen of them in the NPTH group, three in the NPHPT group, and one in the HPHPT group).

## Discussion

While the importance of FGF23 is well-known in phosphate-wasting disorders and in chronic kidney disease, data on FGF23 levels and its regulation in PHPT are not concordant before and after successful parathyroidectomy. Several studies have failed to demonstrate significantly higher FGF23 levels in PHPT patients with normal renal function than in healthy controls as well as any change in FGF23 concentration after parathyroidectomy (12, 16–18). In contrast, other studies showed that increased FGF23 levels observed in PHPT significantly decreased after successful parathyroidectomy (19–22). Furthermore, to our knowledge, FGF23 levels have never been previously evaluated in the increasingly recognized NPHPT phenotype. In the present study, we found that in patients with NPHPT the FGF23 levels are significantly higher than in the controls, albeit to a lesser extent than in patients with HPHPT. In both groups of patients with PHPT, there was a significant positive correlation (more pronounced in the HPHPT group) between PTH and FGF23 levels, as shown in most (18–20), but not in all studies (22). Yamashita H et al. also reported a positive correlation between FGF23 and PTH levels in PHPT patients after regression analysis, only serum calcium, and creatinine clearance appeared to be significant predictors of FGF23 levels (12). We found no significant correlations between FGF23 levels and serum-corrected calcium or 25OHD concentrations in all three study groups. Across the 105 studied patients, there was a significant negative correlation between FGF23 and phosphate levels as well as eGFR, but these correlations remained significant only in the HPHPT patients when the groups were analyzed separately. Several hypotheses have been proposed regarding the mechanisms underlying the development of NPHPT. The multiplicity of putative pathophysiological mechanisms underscores the difficulty to establish a unifying theory regarding the pathogenesis of this disorder. The most prevalent view is that NPHPT is an early form of PHPT which may progress to frank hypercalcemia. Other hypotheses related to partial PTH resistance in bone and kidney or altered parathyroid sensing (23, 24). In a recent study, it was supposed that NPHPT could be a different disease entity, probably more severe compared to mild asymptomatic primary hyperparathyroidism (24). Another hypothesis is that aging and low estrogen status (menopause) play a role in raising PTH levels through a negative calcium balance status (25). Indeed, 93% of the 105 of our study patients were post-menopausal women, and 49% of them were treated for osteoporosis. In our study, contrary to the study of Choi HR et al. (24) we found that patients with NPHPT had a lower prevalence of fractures compared to HPHPT although the bone densitometry scores and bone turnover markers were similar in both groups.

In summary, the pathophysiology of NPHPT has not been clearly elucidated and NPHPT may be a heterogeneous disease. Nevertheless, our data show an incremental rise in FGF23 levels between NPTH, NPHPT, and HPHPT which supports the notion that NPHPT represents an early or milder form of classic PHPT. The positive relationship between FGF23 and PTH, independently of 25OH D levels suggests that the increase in FGF23 may be adaptative to counteract the PTH-induced increase in active vitamin D metabolite.

### Limitations

Our study has several limitations. First, we did not measure the ionized serum calcium. Ionized serum calcium reflects the biologically activated calcium more accurately and directly acts on the CaSR to regulate PTH secretion. Many “normocalcemic” patients are known to have elevated ionized calcium levels (24). Second, we did not measure the level of free 25(OH) D. The value of free 25(OH) D level has been recently highlighted by Wang X et al. (26) who identified that some NPHPT subjects with a normal total of 25(OH)D levels may have secondary hyperparathyroidism based on their low free 25(OH) D levels. However, they compared only 10 NPHPT patients with 20 controls. We neither evaluate the 1,25(OH)_2_D level which is also regulated by FGF23. However, our patients had normal 25(OH) D and eGFR levels, so it is reasonable to assume that their 1,25(OH)_2_D concentration would be normal.

Third, even though our control group included patients with normal calcium and PTH levels, these patients had osteoporosis which may have influenced the calcium/phosphate homeostasis. However, similarly to Fořtová M et al. (22), we did not find any significant difference in bone markers levels or correlation between bone markers and FGF23 levels in the 3 study groups.

### Strengths

Considering that renal function is a major predictive factor of FGF23 levels, the fact that all patients in our study had normal renal function confirms the significant role of FGF23 in PHPT even in its normocalcemic form.

All the subjects in our study were vitamin D repleted, thus we think that our NPHPT patients did not have a secondary hyperparathyroidism.

Finally, the performance of the chemiluminescent assay we used was greater than the ELISA assay used in previous studies. It measures with 100% specificity the whole FGF23 molecule, which represents the biologically active molecule (27).

## Conclusions

FGF23 levels are significantly changed by autonomous PTH hypersecretion. The amplitude of these changes seems to be related to the severity of the PHPT. Further studies are needed to confirm the potential usefulness of FGF 23 as a criterion of progression and severity of PHPT.

## Data availability statement

The raw data supporting the conclusions of this article will be made available by the authors, without undue reservation.

## Ethics statement

The studies involving human participants were reviewed and approved by Helsinki Committee of Emek medical center (EMC 0139-18). The patients/participants provided their written informed consent to participate in this study.

## Author contributions

Each author had substantial contributions to the conception or design of the work; or the acquisition, analysis, or interpretation of data for the work, drafting the work or revising it critically for important intellectual content, providing approval for publication of the content, agreeing to be accountable for all aspects of the work in ensuring that questions related to the accuracy or integrity of any part of the work are appropriately investigated and resolved. All authors contributed to the article and approved the submitted version.
